# Using item response theory as a methodology to impute categorical missing values

**DOI:** 10.1038/s41598-025-20032-7

**Published:** 2025-11-05

**Authors:** Adrienne Kline, Yuan Luo

**Affiliations:** 1 Department of Surgery, Northwestern University, Chicago, USA; 2 Department of Electrical and Computer Engineering, Northwestern University, Chicago, USA; 3 Center for Artificial Intelligence, Northwestern Medicine, Chicago, USA; 4 Department of Preventative Medicine, Northwestern University, Chicago, USA; 5 Institute for Augmented Intelligence in Medicine, Northwestern University, Chicago, USA

**Keywords:** Categorical imputation, Item response theory (IRT), Missing completely at random (MCAR), Missing at random (MAR), Statistics, Computational science, Scientific data

## Abstract

Most datasets suffer from partial or complete missing values, which has downstream limitations on the available models on which to test the data and on any statistical inferences that can be made from the data. Several imputation techniques have been designed to replace missing data with stand in values. The various approaches have implications for calculating clinical scores, model building and model testing. The work showcased here supports using an Item Response Theory (IRT) based approach for categorical imputation, comparing it against several methodologies currently used in the machine learning field including k-nearest neighbors (kNN), multiple imputed chained equations (MICE) and Amazon Web Services (AWS) deep learning method, DataWig. Analyses comparing these techniques were performed on three different datasets that represented ordinal, nominal and binary categories. The data were modified so that they also varied on both the proportion of data missing and the systematization of the missing data. Two different assessments of performance were conducted: accuracy in reproducing the missing values, and predictive performance using the imputed data. Results demonstrated that the proposed method, Item Response Theory for categorical imputation, fared quite well compared to currently used multiple imputation methods, outperforming several of them in many conditions. Given the theoretical basis for the approach, and the unique generation of probabilistic terms for determining category belonging for missing cells, IRT for categorical imputation offers a viable alternative to current approaches.

## Introduction

The purpose of this investigation was to extend the research supporting using Item Response Theory (IRT) models to impute missing data for categorical variables^1,2^ and incorporating it into machine learning contexts that predict outcomes. This is an important additional step insofar as the magnitude of errors in imputed values does not automatically result in a commensurate accuracy differential when using the missing values in subsequent analyses^3^. Imputing missing values for categorical data has proven problematic, much more so than for continuous, normally distributed data^4^. When data include large numbers of categorical data, multiple imputation techniques are challenging, as the space of potential models is enormous^5^. Several attempts to deal with this problem have been introduced, including multinomial and log-linear models^6^, clustering^7,8^ and a variety of multiple imputation methods such as expectation-maximization with bootstrapping, correspondence, latent class analysis, hot deck, and chained equations^9^. Borrowing from psychometric theory, Item Response Theory (IRT) offers a family of models that have been designed specifically to handle categorical data. The process results in a series of probabilities to determine whether the missing value belongs to a particular category. Demonstrating how to leverage these models for use in imputing missing data within machine learning contexts with an outcome variable is the purpose of the current study.

### Missing data

Many datasets suffer from being incomplete, in that they have missing data points in some or all variables. Missing data can occur for many reasons including, but not limited to: hardware limitations (i.e. sensor drop-out), subject loss at follow-up (e.g. patient who did not return or dies), data entry errors, rare events, non-response (i.e. surveys), or the data were intentionally not collected for a case-specific reason. How to best handle missing data can be difficult to resolve, especially when the causal reason for it remains unknown. Even if only a few data points are missing from each variable, the effect of dropout, if performed case-wise, may result in a reduction of power of the statistical test, not having enough data to perform the analysis, or misleading findings if the remaining cohort is not a random sample of all cases. Similarly, many machine learning (ML) models cannot handle missing values, such as support vector machines, GLMnet, and neural networks. The few models that can tolerate missing values are Naive Bayes and some tree-based models under the CART methodology^10^.

Missing data can be classified into three categories^11^; missing completely at random (MCAR), missing at random (MAR) and missing not at random (MNAR). MCAR data follows the logic that the probability of an observation being missing does not depend on observed or unobserved measurements. MAR missing data is conditional on one or more covariates, where the probability of an observation being missing depends only on observed variables in the dataset. Because of the characteristics of MCAR and MAR data, they are amenable to data-driven approaches to handling them. However, when observations are neither MCAR nor MAR, they are classified as MNAR, meaning the probability of an observation being missing depends on unobserved variables/information not available in the analysis. The missing mechanism, then, needs to be theoretically justified and incorporated into the data, as is done, for example, using latent class variable models capable of handling MNAR^12^. Because of the ’top-down’ nature of handling MNAR data, this type of missing data will not be discussed in the current study.

### Traditional imputation techniques

While there is sometimes a focus on prescribing what the appropriate proportion of missing data is, it is more relevant to consider the type of missingness of the data (MCAR or MAR) and the type of imputation used to address the missing data that is of greater import^13^. Interest in both preserving as many cases as possible and using as much of the information in the non-missing data, has led to various methods of imputing values to substitute into the missing cells. Some common examples are forward fill, backward fill, mean or most frequent, and Bidirectional Recurrent Imputation for Time Series (BRITS)^14^. Forward and backward fill work by carrying the most recent value forward or backward, respectively, filling in where appropriate. Imputing with the mean, median or mode works by computing the value of the mean, median or mode in relation to the column and filling this value in where missing. BRITS substitution is specific to time series data. Regression techniques use the information from non-missing variables in the data set to predict the value of the missing data.

### IRT imputation techniques

The concept of IRT for imputation was introduced by Huisman et al.^15^ and was followed up by^16^. However, these studies did not perform a comparison with current state-of-the-art (SOTA) methods, the impact on downstream predictive tasks, or the various algorithmic adaptations required for ordinal, nominal and binary imputation. The purpose of this study is to demonstrate how this technique can be used for imputation and compare its effectiveness with three of the more traditional machine learning multiple imputation methods and how this has impacts downstream machine learning tasks. IRT is a family of mathematical models that link underlying unobserved (latent) traits of individuals/cases to the pattern of their responses to a series of observed variables (i.e., items or features)^17^. This linkage is manifested as one or more logistic functions that specify the probability of obtaining a specific value on any feature as a function of a case’s underlying trait value. These logistic functions are generated using a maximum likelihood iterative approach that analyzes the entire pattern of all feature values for all cases simultaneously. IRT assumes that the latent trait is organized along a continuum called theta ($\theta$) and all individual cases are placed along that continuum. Higher values of $\theta$ are associated with higher levels of the underlying trait. It is assumed that higher values on the features are also associated with higher values of $\theta$.

As part of the analysis process, characteristics of the features, such as their difficulty and discrimination, are estimated as well as an estimate of each case’s standing along the underlying trait – their theta ($\theta$) score. Because IRT mathematical models were developed^18,19^ to link individual responses to test items with test-taker ability, they have primarily been used in the psychological and educational literatures to assess the psychometric properties of items and tests. However, IRT has been used in the machine learning literature to assess the utility of features^20^, natural language processing systems^21^; and classifiers^22,23^.

The current study assesses how well IRT performs as a mechanism for imputation of missing feature data. IRT focuses on the pattern of all the available observed feature values to generate each case’s overall $\theta$ score. Then the imputed missing values are based on each individual case’s $\theta$ score. Because IRT uses all the feature information available for all cases, it is possible to impute valid values for those cases with missing data. One important result, then, of IRT imputed values is that they do not incorporate the outcome variable values in the protocol, as do many other imputation methods. In doing so, IRT avoids the circularity of using the classification outcome to impute missing values. This avoids the problem of overly optimistic findings in predictive modeling studies, when using the outcome to set values for a predictor that is then used to predict that same outcome. Such outcome information would not be available to classify/predict prospective new cases.

Three members of the family of IRT models will be used in the current study. One is the 2-parameter logistic model (2-PL)^24^ used when the features are coded in a binary (0, 1) way. Another is the Graded Response Model (GRM)^25^ used when features have ordinal-level values. Since IRT analyses do not handle continuous interval level data, such data can be converted into multiple ordinal level categories and run using the GRM. The third IRT model is the Nominal Response Model (NRM) used when feature values are nominal/categorical^26^. Salient attributes of these imputation methods are listed in Table 1. Both KNN and MICE typically require categorical variables to be ordinal or be transformed into one-hot encoded if nominal. However, an extension of MICE using predictive mean matching permits handling of binary or ordinal data^27^. MICE is scalable depending on the length of the dataset under consideration. KNN for time series, while theoretically possible, is computationally intractable. Deep learning-based imputation for small datasets is likely, while theoretically and computationally possible, is likely unreliable due to lack of training data.

**Table 1:** Imputation types and attribute comparison.

Attribute	Imputation method			
	KNN	MICE	Datawig	IRT
Categorical imputation	$\checkmark$ $^a$	$\checkmark$ $^a$	$\checkmark$	$\checkmark$
Scalable	x	$\checkmark$ $^b$	$\checkmark$	$\checkmark$
Uses outcome	x	x	$\checkmark$	x
Works for time series	x$^c$	$\checkmark$	$\checkmark$	$\checkmark$
Works for small datasets	$\checkmark$	$\checkmark$	x$^d$	$\checkmark$

## Methods

### Datasets

Three different data sets were selected for this study: Diamonds^28^; Housing^29^ and Heart Disease^30^. These were selected because they: (1) use different types of categorical data to be imputed (ordinal, nominal and binary), (2) allow for comparison of imputing approaches on accuracy and predictive utility, and (3) are complete (no missing values), so the ground truth for the missing cases were available to compare different imputation methods. Thus, they provided a broad comparative field regarding how IRT performs relative to other imputation methods.

Within each data set, a single predictor variable was selected to be missing. Null values were substituted in each of these specified predictor variables in four different amounts (missing 5, 10, 30 and 50%), each following two different structures (MCAR vs MAR). Therefore, each dataset gave rise to eight unique datasets for imputation. To generate the MCAR type data sets, values were randomly replaced with null values. Generating MAR data was performed on a per dataset instance by first identifying a conditional variable on which to generate the MAR data sets. The files were then sorted on the conditional variable and 5, 10, 30 and 50% of the target missing variable was removed from the top of the dataset. To verify MCAR versus MAR missing data structures, Little’s test was used^31^. Little’s test is a modified chi-Square test to determine if one or more systematic relationships between the missing data and other variables exist and is expected to be significant in MAR data sets and non-significant in MCAR data sets. Results are reported in Appendix A, Table S1.

One issue that arose was that since IRT does not accommodate continuous data, such features had to be re-coded into ordinal-level categories, as this is required for use in the GRM analyses. While item responses to continuous data can be imputed using principal components^32^ or confirmatory factor analysis^33^, categorizing continuous variables may offer advantages in modeling non-linear relationships in the imputation process. To do so, histograms of the data were generated for each continuous feature and cut points made to preserve the original shape of the distribution, as many of the feature variables were non-normally distributed. Data that were affected in such a way were split into quartiles, providing four-level ordinal variables. This conversion was only done when running the IRT imputations.

#### Ordinal imputation dataset

The diamonds data is a set of 53,920 diamond cases with a continuous outcome (price). The eight features are a combination of ordinal (e.g., clarity) and continuous (e.g., dimensions along x, y, z). The feature that was selected to be missing for purposes of this study was color (an ordinal variable with 8 different levels). Other variables included price in US dollars ($326-$18,823), carat weight of the diamond (0.2-5.01), cut quality of the cut (Fair, Good, Very Good, Premium, Ideal), color; from J (worst) to D (best), clarity; (I1 (worst), SI2, SI1, VS2, VS1, VVS2, VVS1, IF (best)), x length in mm (0-10.74), y width in mm (0-58.9), z depth in mm (0-31.8), depth total depth percentage = z / mean(x, y) = 2 * z / (x + y) (43-79) and table width of top of diamond relative to the widest point (43-95). The criterion measure was the continuous variable of Price in U.S. dollars. A list of the variables and their codes are shown in Table 2. The conditional variable to generate the MAR data sets was ‘carat size’ in this data set.

**Table 2:** Diamond dataset.

Feature	Feature type
Carat	Numeric-continuous
Cut	Continuous
Color	Ordinal
Depth	Numeric-continuous
X (mm)	Numeric-continuous
Y (mm)	Numeric-continuous
Z (mm)	Numeric-continuous
Price (outcome)	Numeric-continuous

#### Nominal imputation dataset

The housing data set is made up of 10,692 unique rental units and their features. The continuous criterion measure was rental price in Brazilian Real. Other features included whether the space was furnished or not, number of rooms, square footage, number of bathrooms and the city in which it was located. The feature that was selected to be missing for purposes of this study was city (a nominal categorical variable with 5 unique values). The conditional variable to generate the MAR data sets was ’number of rooms’ in this data set. The variables and their feature types can be seen in Table 3.

**Table 3:** Housing dataset.

Feature	Feature type
City	Categorical-nominal
Area	Numeric-continuous
Rooms	Numeric-continuous
Bathrooms	Numeric-continuous
Furniture	Numeric-binary
Rent price (outcome)	Numeric-continuous

#### Binary imputation dataset

The heart disease data is a set of 253,680 responses from Behavioral Risk Factor Surveillance System (BRFSS) 2015, generated by the CDC to be used for the binary classification of heart disease/attack. The criterion measure was binary (no heart disease - coded 0, heart disease - coded 1). 23,893 of the cases had heart disease. An equivalent number were randomly selected from the non-heart disease cases, producing a final and balanced data set of 47,786 cases. A list of the variables and their codes are shown in Table 4. The feature that was selected to be missing for purposes of this study was high blood pressure (a binary variable). The conditional variable to generate the MAR data sets was ‘age’ in this data set.

**Table 4:** Heart disease dataset.

Feature	Feature type
BMI	Numeric-continuous
Age	Numeric-continuous
Smoker	0,1 - binary
Stroke	0,1 - binary
Diabetes	0,1 - binary
No physical activity	0,1 - binary
No vegetables	0,1 - binary
Difficulty walking	0,1 - binary
High cholesterol	0,1 - binary
High blood pressure	0,1 - binary
Heart disease or attack (outcome)	0,1 - binary

### Imputation methods

#### Existing methods

Three commonly used, robust imputation methods were employed in this study, k-NN, MICE, and a deep learning method called DataWig. Three IRT models were used to impute the values of the binary, ordinal and categorical data sets. K-NN works very much like the algorithm for classification. The substituted value is based on a specified number ’k’ of the closest point estimates in an n-dimensional space. MICE also known as Sequential Regression Imputation, was developed by Rubin^34^ and leverages a series (chain) of regression equations to obtain imputation values. MICE starts with a simple imputation method, such as mean substitution. However, the process is repeated several times on different portions of the data and regressed on other variables, where the final imputed value converges to a stable solution. DataWig is a deep learning imputation method developed by Amazon Web Services (AWS)^35^ that uses a Long Short Term Memory network (LSTM). It follows a similar approach as that of MICE that can be extended to allow for different types of data (categorical, numerical, text) to be used when imputing missing values. For categorical variable imputation, an EmbeddingFeaturizer is used, where training data are comprised of rows of complete data and the training supplies the remaining structured dataset. The predicted outcome is the value to be imputed and is subsequently substituted into the final dataset. It should be noted that both MICE and DataWig have inherent randomness as part of the underlying imputation algorithms and thus repeated imputed datasets (5 for each) and their standard errors have been created for these methodologies throughout the results presented below.

In our imputation framework for MICE, we enabled posterior sampling via a (non-linear) Gaussian process regressor so that, for each variable with missing observations, the imputed values are not fixed at the model’s conditional mean but are instead drawn from the fitted regression’s full posterior predictive distribution. By sampling rather than simply plugging in point estimates, we explicitly propagate the uncertainty inherent in each conditional model into the imputed dataset. This yields a collection of plausible, complete datasets whose between-dataset variability faithfully reflects the underlying uncertainty in the missing values. The within imputation variance is recorded in the result tables below. Our deep learning imputation framework for Datawig was comprised of a 75% and 25% test set. The hyperparameter search space comprised of varying the learning rate, number of epochs and number of latent dimensions, where the best model was selected (learning rate: 0.04, epochs: 100, early stopping patience: 5 epochs, and batch size: 16). The model leveraged for imputation was implemented separately on the training and test sets to prevent data leakage. In our imputation framework for k-NN-based imputation, hyperparameters for the number of neighbors were permitted to vary (3,5,7,10) where the highest performing imputation with respect to the accuracy of the imputed value (rather than predictive performance) was retained.

### IRT imputation

IRT provides an alternative approach to imputation, as described earlier. The IRTPRO (Vector Psychometric Group, 2021) program was used to estimate the IRT feature and case parameters in all data sets. Data can be imported into the program from a number of different file formats, including the type used in this study (.csv). All missing data cells were coded with -1. The interface allows for a mixture of different types of features within the same analysis (i.e., a mix of binary, ordinal, or categorical features can be used in the same analysis). Each model was specified to be based on one group of cases using a unidimensional set of features. IRTPRO uses marginal maximum likelihood^36^ to estimate feature parameters and expected a posteriori (EAP) to generate a $\theta$ score for each case. Parameters are estimated in the logistic metric. Some programs have historically rescaled the parameters to approximate the normal to give function, but this is not done in IRTPRO as has been suggested more recently^37^. Three models were specified: 1) Two Parameter Logistic (2-PL) (binary) for the Heart Disease dataset, 2) Graded Response Model (GRM) (ordinal) for the Diamonds dataset and 3) Nominal Response Model (NRM) (nominal categories) for the Housing dataset.

#### 2-PL model

In the case of a binary response (0, 1) using these estimated parameters, the linking function between the underlying trait and particular feature can be described as follows (Eq 1):

1 $$\begin{aligned} Pij(U_{ij}=1\mid \theta ) = \frac{e^{a_{i}(\theta - b_{ij})}}{1+ e^{a_{i}(\theta - b_{ij})}} \end{aligned}$$

**Fig. 1:**
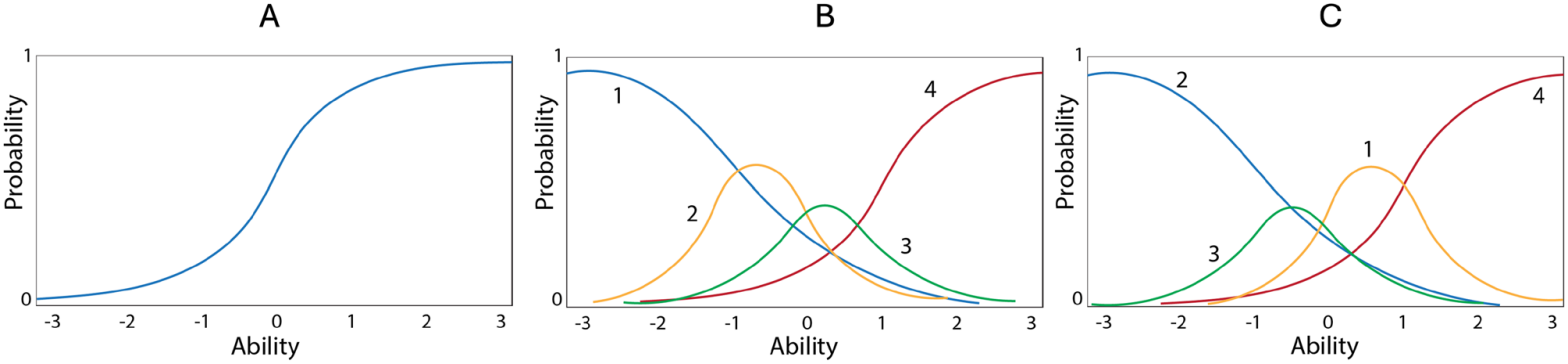
(**A**) 2-parameter logistic model (2-PL) (**B**) Graded response model (GRM) (**C**) Nominal response model (NRM).

The model for binary variables in equation 1 has the simple interpretation of success being equal to the value of the person parameter $\theta$ relative to the value of the item parameters. The probability of being in the “1” category on a particular item *i* can be ascertained for any case with a specific $\theta$-value. Using this model, a missing binary variable can be imputed - cases with probabilities below 50% are imputed as 0 and those with probabilities above 50% are imputed as 1. Figure 1A showcases the curve for this model, where ability $\theta$ is a row/case characteristic and parameter values are associated with the variable (item).

#### GRM

The graded response model (GRM) represents a family of mathematical models that deals with ordered polytonomous categories, the curves for the 5 different options can be seen in Fig. 1B. It uses a two-step process to link the trait to features^25^. In the first step, a series of 2-PL functions for each of the category option boundaries are generated. For example, if one has a 5-option feature (coded 0, 1, 2, 3, 4), there would be 4 boundary functions: above 0 but less than 1, above 1 but less than 2, above 2 but less than 3 and above 3 but less than 4. In this first step, threshold parameters for each of the features’ option boundaries and an overall slope parameter for the feature are generated. If $\theta$ is the latent ability, and $U_i$ is a random variable to denote the graded item response to item i, and $u_i=(0,1,...,m_i)$ denotes the actual responses. The category response function, $P_{ui}(\theta )$, is the probability with which an examinee with ability $\theta$ receives a score $u_i$ is:

2 $$\begin{aligned} P_{ui}(\theta )\equiv P[U_i=u_i \mid \theta ] \end{aligned}$$

Probabilities based on the other combinations, given $\theta$, are computed by subtracting the adjacent $P^*_{ik}(\theta )$:

3 $$\begin{aligned} P^*_{ik}(\theta ) = P^*_{ik}(\theta ) - P^*_{ik+1}(\theta ) \end{aligned}$$

Therefore, in expanding Eq. 3 for a 5 category GRM, we would get:

4 $$\begin{aligned} \begin{aligned} Option 0: P_{i0}(\theta )&= 1.0 - P_{i1}(\theta )\\ Option 1: P_{i1}(\theta )&= P_{i1}(\theta ) - P_{i2}(\theta )\\ Option 2: P_{i2}(\theta )&= P_{i2}(\theta ) - P_{i3}(\theta )\\ Option 3: P_{i3}(\theta )&= P_{i3}(\theta ) - P_{i4}(\theta )\\ Option 4: P_{i4}(\theta )&= P_{i4}(\theta ) - 0.0 \end{aligned} \end{aligned}$$

Continuing with the example of 5 categories the dichotomies would refer to the probability of being coded 1: (1) in category 0 contrasted with categories 1, 2, 3, and 4; (2) in categories 0, 1 contrasted with categories 2, 3, and 4; (3) in categories 0, 1, 2 contrasted with categories 3 and 4; 4) in categories 0, 1, 2, 3 contrasted with category 4.

The second step of the process uses subtraction between the probabilities for each option boundary of that feature to estimate the probabilities for each option. The probability of responding at the lowest option or above is 1.0, and the probability of responding above the highest alternate is 0.0. Using this model, the missing ordinal cells were imputed and categories assigned for each case based on the category with the highest probability.

#### NRM

The nominal response model (NRM) also uses a two-step process (divide-by-total) to link the ability with features^38^. In a typical nominal response model, a person N responds to each of n items, where the item *i* admits responses in $m_i$ mutually exclusive categories, as in the case with a multiple-choice exam. The curves for such a model are presented in Fig. 1C. In the first step, functions for each of the category options are generated by estimating the slopes *a* and intercepts *c* for each option. Based on a case’s score, the probability of being coded “1” on a particular category *j* of a feature *i* is calculated as the ratio of the probability of being in that category divided by the sum of the probabilities of falling into any of the categories on that feature, see Eq. 5.

5 $$\begin{aligned} P_{ij}(\theta ) = \frac{\exp (a_{ij}\theta + c_{ij})}{\sum _{x=0}^m \exp (a_{ij}\theta + c_{ij})} \end{aligned}$$

To ensure model identification in NRM, one of two constraints must be set for parameter estimation. Either the sum across feature slopes and feature intercepts must be set to zero ($\theta a_{ij} = \theta$, $c_{ij} = 0$), or the lowest response category for each feature must be set to zero ($a_{i1}$ = $c_{i1}$ = 0). The IRTPRO program opts for the latter of these two constraint options, as has been suggested to be more plausible^39^. As with the GRM, this analysis estimates the category into which the case is most likely to fall. For imputation, each category was calculated based on parameters $a_i$ and $c_i$ and ability $\theta$ , and the category with the highest probability was assigned the imputed value for nominal level data.

### Assessments

The first assessment was to test whether the MCAR and MAR manipulations conformed to expectations. Next, the direct accuracy of imputation was assessed. It is common to only assess the impact of imputation on missing values on the downstream results of predictive accuracy of the entire dataset. This makes sense insofar as these results have the most relevance for ultimate use. However, we also provide an assessment of the accuracy of the imputed missing values themselves as a methodological check, as the results may provide insight into the later ultimate performance of the imputation method used. The last assessment was to assess the predictive utility of the data. Possible differences between the imputation methodologies (4 levels) and type of missing data (2 levels) on the effects on the accuracy of imputation and the predictive utility of imputed datasets were examined. Factorial Analyses of Variances and follow-up tests using a Bonferroni correction were used for these analyses. Effects were considered significant at *p*<.01, given the number of tests that were conducted.

#### Direct accuracy assessment

To assess the imputations relative to the complete datasets, F1 scores (Eq. 6) were calculated for the cells that had been imputed. The F1 statistic was chosen for several reasons. First, we had a ground truth value with which to assess accuracy. Second, the imputed data points are discrete. Third, it was assumed that there was an imbalance in the actual values of the missing cells. Fourth, IRT does not use multiple imputation so a variance on this cannot be ascertained. Fifth, F1 provides a single, easily interpretable value ranging from 0 to 1, with higher values indicating better accuracy and is used in assessing the accuracy of missing values imputation.

6 $$\begin{aligned} F1 = 2*\frac{precision*recall}{precision + recall} \end{aligned}$$

#### Predictive utility assessment

Machine learning models were trained to compare relative predictive utility between the different imputation methods with the original complete data (ground truth).Several machine learning methods were trialed on the original data sets, which included Linear Regression, Bayesian Ridge regression, Random Forest Regressor, and XGBoostRegressor for the regression outcome data sets (Diamonds and Housing). Random Forest, neural network (NN), support vector machine (SVM) and XGBoost algorithms were used for classification outcome (Heart Disease Data set). Hyperparameters were determined using a random search within the various algorithms. The best model for each dataset was determined using the original dataset and then used with the imputed datasets to allow for a consistent comparison.

Root Mean Square Error (RMSE) summary values for the Diamond and Housing outcome predictions were used to assess the fit of the expected to observed values, where lower values are better. Area Under the Curve (AUC) was used to assess the models’ capability of distinguishing between classifications for the Heart Disease outcome predictions, where higher values are better.

## Results

### Testing imputed values accuracy (F1)

Tables 5, 6 and 7 show the F1 values across imputed missing cells.

**Table 5:** F1 values following imputation of diamond dataset, stratified by type and amount missing.

Missing type	Num. missing	KNN	MICE$^1$ ± Std. err	Datawig$^1$ ± Std. err	IRT
MAR	2696	0.192	0.161 ± 0.003	0.192 ± 0.007	0.289
MAR	5392	0.201	0.159 ± 0.001	0.207 ± 0.002	0.238
MAR	16,176	0.199	0.162 ± 0.001	0.202 ± 0.003	0.166
MAR	26,960	0.185	0.153 ± 0.0005	0.191 ± 0.008	0.185
Marginal means		0.194	0.159	0.198	0.220
MCAR	2696	0.231	0.164 ± 0.003	0.222 ± 0.002	0.208
MCAR	5392	0.223	0.158 ± 0.002	0.218 ± 0.001	0.213
MCAR	16,176	0.219	0.159 ± 0.001	0.217 ± 0.002	0.212
MCAR	26,960	0.219	0.157 ± 0.001	0.221 ± 0.001	0.209
Marginal means		0.223	0.160	0.220	0.211

**Table 6:** F1 values following imputation of housing dataset, stratified by type and amount missing.

Missing type	Num. missing	KNN	MICE$^1$ ± Std. err	Datawig$^1$ ± Std. err	IRT
MAR	535	0.328	0.203 ± 0.008	0.523 ± 0.0004	0.523
MAR	1069	0.332	0.206 ± 0.008	0.526 ± 0.0002	0.527
MAR	3208	0.298	0.204 ± 0.004	0.515 ± 0.005	0.522
MAR	5346	0.187	0.198 ± 0.002	0.438 ± 0.004	0.456
Marginal means		0.286	0.203	0.501	0.507
MCAR	535	0.263	0.197 ± 0.005	0.566 ± 0.003	0.563
MCAR	1069	0.264	0.193 ± 0.005	0.545 ± 0.0004	0.544
MCAR	3208	0.255	0.194 ± 0.003	0.549 ± 0.0008	0.550
MCAR	5346	0.258	0.194 ± 0.002	0.546 ± 0.0006	0.553
Marginal means		0.260	0.195	0.551	0.553

**Table 7:** F1 values following imputation of heart disease dataset, stratified by type and amount missing.

Missing type	Num. missing	KNN	MICE$^1$ ± Std. err	Datawig$^1$ ± Std. err	IRT
MAR	2389	0.852	0.662 ± 0.005	0.866 ± 0.001	0.842
MAR	4779	0.798	0.632 ± 0.003	0.836 ± 0.0003	0.798
MAR	14,336	0.664	0.579 ± 0.002	0.753 ± 0.001	0.720
MAR	23,893	0.655	0.565 ± 0.0003	0.564 ± 0.015	0.691
Marginal means		0.742	0.610	0.755	0.763
MCAR	2389	0.699	0.570 ± 0.008	0.734 ± 0.001	0.709
MCAR	4779	0.686	0.575 ± 0.003	0.732 ± 0.0008	0.719
MCAR	14,336	0.691	0.574 ± 0.002	0.729 ± 0.0005	0.716
MCAR	23,893	0.697	0.574 ± 0.001	0.729 ± 0.0003	0.713
Marginal means		0.693	0.726	0.730	0.714

In the Diamond dataset, the ordinal variable (5-levels) of ’color’ category was imputed. There was a significant main effect of methodologies collapsed across MAR and MCAR data sets (F(3,24)=13.3, *p* < 0.001). Follow-up tests showed that KNN, DataWig, and IRT all performed significantly better than MICE in reproducing the missing values. Overall, the F1 value for this data set across all methodologies was 0.20, indicating that this is a difficult imputation task.

In the Housing dataset, where the imputed variable was a nominal categorical variable, there was also a main effect of methodologies collapsed across MAR and MCAR data sets (F(3,24)=243.35, *p* < 0.001). Follow-up tests showed that MICE performed significantly poorer than KNN, DataWig and IRT; KNN performed significantly poorer than DataWig and IRT. DataWig and IRT performed similarly. Overall, the F1 value for this data set across all methodologies was 0.38, indicating that this is not as difficult a task as an ordinal categorical imputation, but is still difficult.

In the Heart Disease dataset, where the imputed variable was a binary category, there was again a main effect of methodologies collapsed across MAR and MCAR data sets (F(1,24)=9.38, *p* < 0.001). Follow-up tests showed that MICE performed significantly poorer than KNN, DataWig and IRT. Overall, the F1 value for this data set across all methodologies was 0.70, indicating that this imputation task is a relatively easy one. Performing a visual inspection of Tables 5, 6 and 7, increasing the percentage of missing items consistently negatively impacts the F1 scores when the items are missing at random (MAR).

### Effects on machine learning outcomes

XGBoostRegressor and XGBoost machine learning algorithms outperformed others tested and were used in the regression (Diamonds and Housing datasets) and classification (Heart Disease) analyses, respectively. The results are reported in Tables 8, 9 and 10. At the bottom of each table is the recorded performance of the full (no missing) data sets.

For the Diamond data set, there was a significant classification main effect of: (1) imputation methodology collapsed across MAR and MCAR data sets (F(3,24)=9.20, *p* < 0.001) and (2) missing data type collapsed across methodology (F(1,24)=16.67, *p* < 0.001), and an interaction effect of imputation by missing data (F(3,24)=11.69, *p* < 0.001). Post-hoc tests of the interaction showed that the effect was due to the poor performance of DataWig with the MCAR data set. The RMSE for the original, non-missing Diamond data was 0.22, while the average overall RMSE across all imputations was 0.23 for the MAR and 0.34 for the MCAR, indicating that imputing missing data in the MCAR imputation situation had a negative effect on the predictive model.

For the House data set, there was again a significant classification main effect of: (1) imputation methodology collapsed across MAR and MCAR data sets (F(3,24)=12.40, *p* < 0.001) and (2) missing data type collapsed across methodology (F(1,24)=13.88, *p* < 0.001), and an interaction effect of imputation by missing data (F(3,24)=9.49, *p* < 0.001). Post-hoc tests of the interaction showed that in the MAR data KNN and IRT outperformed MICE. In the MCAR data IRT outperformed KNN and MICE. The KNN imputation method produced poorer results with the MCAR data than the MAR data. The RMSE for the original, non-missing House data was 0.44, while the average overall RMSE across all imputations was 0.61 for the MAR and 0.68 for the MCAR, indicating that imputing missing data in all imputation situations had a negative effect on the predictive model.

There were no classification effects of imputation methodology or missing data type in the Heart Disease data set. The RMSE for the original, non-missing Heart Disease data was 0.83, and the average overall RMSE across all imputations was 0.83 for the MAR and 0.83 for the MCAR, indicating that imputing missing data did not affect the performance on the predictive model in the binary data situation.

**Table 8:** RMSE for diamond dataset.

Type	Missing (%)	KNN	MICE$^1$ ± Std. err.	Datawig$^1$ ± Std. err.	IRT
MAR	2696 (5)	0.224	0.224 ± 0.00001	0.216 ± 0.00005	0.280
MAR	5392 (10)	0.223	0.224 ± 0.00002	0.216 ± 0.00003	0.280
MAR	16,176 (30)	0.223	0.225 ± 0.00002	0.215 ± 0.0002	0.278
MAR	26,960 (50)	0.222	0.227 ± 0.00001	0.214 ± 0.0002	0.267
Marginal means		0.223	0.225	0.215	0.276
MCAR	2696 (5)	0.230	0.232 ± 0.0001	0.373 ± 0.0004	0.283
MCAR	5392 (10)	0.234	0.238 ± 0.0002	0.473 ± 0.0004	0.283
MCAR	16,176 (30)	0.249	0.258 ± 0.0002	0.704 ± 0.0006	0.285
MCAR	26,960 (50)	0.262	0.270 ± 0.0002	0.836 ± 0.0015	0.286
Marginal means		0.244	0.250	0.597	0.284
Original data		RMSE:	0.2241

**Table 9:** RMSE for housing dataset.

Type	Missing (%)	KNN	MICE$^1$ ± Std. err.	Datawig$^1$ ± Std. err.	IRT
MAR	535	0.496	0.692 ± 0.0107	0.635 ± 0.0084	0.488
MAR	1069	0.444	0.737 ± 0.0294	0.587 ± 0.0100	0.614
MAR	3208	0.606	0.678 ± 0.0009	0.586 ± 0.0034	0.607
MAR	5346	0.582	0.695 ± 0.0161	0.610 ± 0.0112	0.641
Marginal means		0.532	0.701	0.605	0.587
MCAR	535	0.760	0.745 ± 0.0023	0.591 ± 0.0101	0.445
MCAR	1069	0.752	0.749 ± 0.0026	0.670 ± 0.0037	0.645
MCAR	3208	0.796	0.768 ± 0.0082	0.625 ± 0.0072	0.455
MCAR	5346	0.809	0.806 ± 0.0059	0.765 ± 0.0048	0.554
Marginal means		0.779	0.767	0.663	0.525
Original data		RMSE:	0.4380

**Table 10:** AUC for heart disease dataset.

Type	Missing (%)	KNN	MICE$^1$ ± Std. err.	Datawig$^1$ ± Std. err.	IRT
MAR	2389	0.832	0.831 ± 0.0001	0.831 ± 0.0001	0.830
MAR	4779	0.830	0.830 ± 0.0002	0.831 ± 0.0002	0.832
MAR	14,336	0.827	0.828 ± 0.0001	0.829 ± 0.0003	0.827
MAR	23,893	0.827	0.827 ± 0.0003	0.828 ± 0.0002	0.829
Marginal means		0.829	0.829	0.830	0.829
MCAR	2389	0.829	0.831 ± 0.0003	0.832 ± 0.0004	0.832
MCAR	4779	0.831	0.830 ± 0.0005	0.831 ± 0.0002	0.831
MCAR	14,336	0.829	0.828 ± 0.0005	0.829 ± 0.0002	0.828
MCAR	23,893	0.828	0.826 ± 0.0003	0.828 ± 0.0002	0.829
Marginal means		0.829	0.829	0.830	0.830
Original data AUC:		0.8313			

## Discussion

The results suggest that IRT-based imputation is a viable alternative to some of the more established methods for categorical imputation. We specifically examined the direct accuracy of the imputed values themselves in relation to the ground truth of those values in addition to examining the effects on predictive accuracy. This is a somewhat unorthodox step and is not always conducted given that the predictive accuracy of the imputation technique is of most relevance. However, a check on this methodological step of the process has been suggested by others as being worthwhile^40–43^, and may provide insights how this might affect predictive outcomes.

IRT returned more accurate values than MICE for the Diamond data (ordinal), and for the Heart Disease data (binary), and more accurate values than KNN and MICE for the Housing data (nominal). For the imputation accuracy there were no effects of missing data type. It did not seem to matter whether there was any structure (MAR) or not (MCAR) in the ‘missingness’ of the data that would systematically impact the type of imputation used.

In terms of the predictive utility of these substitutions, DataWig was significantly poorer than all the other imputation methodologies with the MCAR data for the Diamond (ordinal) data. In the Housing data (nominal) IRT was superior to MICE in the MAR data and superior to KNN and MICE for the MCAR data. In no instance was IRT significantly poorer that the other methods. For the predictive accuracy there were effects of missing data type for the ordinal and nominal substituted data. MCAR data were usually predicted more poorly (other than for one instance of IRT) than the MAR data. This makes sense insofar as the methodologies in MAR are utilizing other information in the data sets to impute missing values. A quite important finding was that the imputing missing data values adversely affected the performance of the predictive models, particularly with the nominal data as well as the MCAR data in the ordinal situation. This should give researchers pause to carefully consider the most viable imputation option for their own research question when their data contain such missing data.

While the amount of missing data was manipulated, there did not seem to be a very large effect of this variable on the results and was not tested empirically. The one exception was the predictive utility of the Diamond data when MCAR missing data were imputed by DataWig; as the proportion of missing data increased, there was obviously an impediment to the features’ overall predictive utility.

One notable finding was that the ordinal categorical data were most difficult for all imputation techniques, followed by the nominal imputations, with the binary imputations most easily addressed. This intriguing finding is quite possibly a result of the one-hot encoding limitation required by algorithms such as KNN and MICE, and distributional effects of ordinal categories. Binary imputation with two distinct classes leaves fewer available options, and thus being correct by chance is higher as a result.

There were no effects of imputation on high blood pressure (binary) from the heart disease data (binary missing data), indicating that none were superior/inferior with this type of data, with accuracy or predictive utility. This may be due to blood pressure existing in 2 distinct states. On closer examination of the heart disease data there is a substantial imbalance; 23,893 blood pressure values were missing (MCAR: 1-13,694, 0-10,209) and (MAR: 1-16,660, 0-7,233), where ’1’ denotes high blood pressure and ’0’ does not. However, results were very similar.

Although DataWig is often described as being superior to other imputation methods in that it handles different types of data, it did not perform as well as the others in this study on some data sets – more poorly on the ordinal data than all the others, no better than IRT on the nominal data, and no better than any of the others on the binary data. There is also the circularity issue in using DataWig as it uses the outcome variable when estimating missing values. As per DataWig’s documentation, it requires at least 10 times more rows than the unique number of categories to impute missing values for categorical variables. In the current study, it had difficulty imputing a category that appeared infrequently within a categorical variable.

Although not shown here, a strength of IRT for categorical imputation is when continuous feature values have a non-linear relationship to the outcome, or are highly skewed, modifying the variable to be a categorical estimate may be a very useful alternative. For instance, many lab values in healthcare data are associated with poor health outcomes if they are ’out of range’ - abnormally high or abnormally low. Hypo- and hypernatremia are examples of this. These pose a unique challenge for linear imputation methods. Employing IRT for categorical imputation, cut points could be made that delineate the normal range (135-145 mmol/L) from abnormally high or abnormally low. Missing values could be imputed under GRM or NRM methodology in IRT. In addition, IRT for categorical imputation methods can be used with supervised or unsupervised data sets.

The IRT for categorical imputation method was tested on multiple data sets, but there are limitations to the work. One was that the data from a single variable was missing; this was done to control the effects. It is possible that if the structure of the missing data was modified, the results might change. This remains an open invitation to researchers in other disciplines to control, as our study did, as many variables as possible to ensure the internal validity of the findings. Another is that only three different datasets were used in the study. While this is true, the purpose of the study was to introduce the IRTCI method and to show its effects it on different types of categorical-level data (binary, nominal and ordinal). While this does limit the generalizability of the findings, the analyses conducted did demonstrate a fairly comprehensive comparison of imputation approaches. Third, IRT for categorical imputation requires the movement between two different software platforms, and moving between them can be a deterrent. Fourth, IRT for categorical imputation is useful primarily for categorical imputations (binary, nominal, and ordinal) as demonstrated in this study. Another opportunity for future research into this method includes adapting it for use with continuous features. IRT protocols allow for categorization of continuous data into many ordinal-level groups (e.g., 10–15). Such a set would be a ’near continuous’ approximation of the data. Imputed missing values could be mapped back to the distribution from whence it came, allowing for a point estimate of the data. Such an approach would require large data sets to ensure adequate numbers of cases in each group. Additional future work is warranted to demonstrate how this method would perform. Lastly, IRT makes some restrictive assumptions about the data. The first is that the latent trait is organized along a continuum $\theta$ and all individual cases are placed along that continuum. Higher values of $\theta$ are associated with higher levels of the underlying trait. It is assumed that higher values on the features are also associated with higher values of $\theta$. Another is that the items are locally independent. This means that each item/measure is independent of the others, other than for the underlying latent trait. There is an additional assumption for the purposes of this study: that the underlying “trait” is unidimensional. Violations of these assumptions imply that using unidimensional IRT models are not appropriate.

Our findings support the use of the IRT-based categorical imputation method that is of particular importance in machine learning contexts. Categorical imputation poses some unique problems, unlike multiple imputation based on continuous, normally distributed data; categorical multiple imputations with many variables result in large numbers of higher order interactions^4^. Most imputation methods used in machine learning require transformation to one-hot encoded values and do not have native methods for handling nominal categories. In addition, the use of the outcome variable in the estimation process biases these imputation processes. In contrast, our technique uses a theoretically justified probabilistic approach to imputing the most likely value for a categorical variable. As it is outlined in this study, IRT for categorical imputation presents a viable alternative to existing methods.

## Supplementary Information

Supplementary Information.

## Data Availability

The diamond data set can be accessed at URL: https://www.kaggle.com/datasets/shivam2503/diamonds (accessed 02.13.2022). The housing data set can be accessed at URL: https://www.kaggle.com/datasets/amirmohammadparvizi/houses-to-rent (accessed 02.13.2022) The heart disease data set can be accessed at URL: https://www.kaggle.com/datasets/alexteboul/heart-disease-health-indicatorsdataset (accessed 02.13.2022).
