# Supplementary material for: Using item response theory as a methodology to impute categorical missing values
Source: Sci Rep. 2025 Nov 5;15:38675. doi: 10.1038/s41598-025-20032-7 (PMC12589431; doi:10.1038/s41598-025-20032-7)
Supplement: Supplementary file 1 — Supplementary Information. [file 41598_2025_20032_MOESM1_ESM.pdf]

Appendix A: Tests of the MCAR and MAR data set assumptions

Appendix Table S1 showcases the results after performing Little's test to ensure each dataset was created in a MAR and MCAR fashion and in varying amounts (5,10, 30, and 50%). As can be seen in the last column of Table 5 the test statistics are significant when MAR data were created as conditional on another column and not significant when values were removed at random. These results are in accordance with the ultimate goal of being able to compare how data being either MAR or MCAR influences imputed methodologies.

Table S1. Little's Test Results for Datasets

Dataset	Missing type	% missing	Num. instances missing	test stat, (p-value)
Diamond	MAR	5	2696	10,693 (0.000)
Diamond	MAR	10	5392	18,065.77 (0.000)
Diamond	MAR	30	16176	41,009.07, (0.000)
Diamond	MAR	50	26960	41,409.31, (0.000)
Diamond	MCAR	5	2696	1.899, (0.984)
Diamond	MCAR	10	5392	6.222, (0.622)
Diamond	MCAR	30	16176	7.472, (0.487)
Diamond	MCAR	50	26960	5.258, (0.730)
Housing	MAR	5	535	964.022, (0.000)
Housing	MAR	10	1069	2017.706, (0.000)
Housing	MAR	30	3208	5451.812, (0.000)
Housing	MAR	50	5346	7284.074, (0.000)
Housing	MCAR	5	535	1.307, (0.934)
Housing	MCAR	10	1069	1.087, (0.955)
Housing	MCAR	30	3208	1.867, (0.867)
Housing	MCAR	50	5346	1.833, (0.872)
Heart Disease	MAR	5	2389	14376.354, (0.000)
Heart Disease	MAR	10	4779	22067.449, (0.000)
Heart Disease	MAR	30	14336	31409.706, (0.000)
Heart Disease	MAR	50	23893	30324.046, (0.000)
Heart Disease	MCAR	5	2389	16.019, (0.099)
Heart Disease	MCAR	10	4779	14.994, (0.132)
Heart Disease	MCAR	30	14336	15.230, (0.124)
Heart Disease	MCAR	50	23893	5.566, (0 .850)
